# Relationship between surgical field contamination by patient's exhaled air and the state of the drapes during eye surgery

**DOI:** 10.1038/s41598-023-31926-9

**Published:** 2023-04-07

**Authors:** Masakazu Morioka, Yoshihiro Takamura, Hideki T. Miyazaki, Makoto Gozawa, Yutaka Yamada, Ryohei Komori, Kengo Tanaka, Masaru Inatani

**Affiliations:** 1 Department of Ophthalmology, Faculty of Medical Sciences, University of Fukui, Yoshida, Japan; 2 National Institute for Materials Science (NIMS), Tsukuba, Japan

**Keywords:** Retinal diseases, Bacterial infection

## Abstract

The coronavirus disease (COVID-19) pandemic has led to a dramatic increase in facemask use. Consequently, it has been reported that exhaled airflow toward the eyes can cause the dispersal of bacteria into the eyes, potentially increasing the incidence of postoperative endophthalmitis. In addition to wearing a facemask, gaps between the surgical drape and skin can also direct exhaled airflow toward the eyes. Here, we aimed to examine how the risk of contamination varies depending on the state of the drapes. We used a carbon dioxide imaging camera to visualize changes in exhaled airflow under different drape conditions and a particle counter to evaluate changes in the number of particles around the eye. The results revealed airflow present around the eye and a significant increase in the number of particles when the nasal side of the drape was detached from the skin. However, when a metal rod called “rihika” was used to create space above the body, the airflow and number of particles were significantly reduced. Thus, if drape coverage becomes incomplete during surgery, exhaled airflow toward the eye may contaminate the surgical field. On hanging up the drape, airflow can escape in the direction of the body, potentially preventing contamination.

## Introduction

The global coronavirus disease (COVID-19) pandemic caused by severe acute respiratory syndrome coronavirus 2 (SARS-CoV-2) started in early 2020^1^. COVID-19 is primarily a respiratory disease that can manifest as an acute upper and/or lower respiratory tract syndrome of varying severity. By the end of June 2022, more than 500 million people had contracted the infection worldwide, and 6 million died^2^. The main routes of infection are droplet and contact transmission, and wearing a mask effectively prevents the spread of the infection^3,4^.

Consequently, the rate of facemask use in daily life has increased dramatically worldwide compared to the pre-pandemic rate. In this context, ophthalmologists are keenly interested in the effects of airflow from the mouth to the eyes. The main concern is that the exhaled airflow and droplets generated between the skin and facemask may get expelled toward the eyes and induce various eye diseases. The dispersal of bacteria from the oral cavity into the eye may contaminate the ocular surface and increase the incidence of bacterial endophthalmitis^5^. Bacterial endophthalmitis is a complication caused by bacterial infection after an intravitreal injection or endophthalmic surgery. The frequency of endophthalmitis has been reported to range from 0.01% to approximately 0.26%, 10%, and 0.11% after injection, cataract surgery, and vitrectomy, respectively^6–9^. Although endophthalmitis is a relatively rare complication, it can cause severe visual impairment. The causative organisms are usually *Staphylococcus spp.* commonly detected on the ocular surface; however, oral bacteria have occasionally been detected as the causative organisms, and the prognosis in such cases is reported to be poor^10^. Some reports have indicated that bacteria were detected in a bacterial medium placed in front of the forehead of individuals coughing or talking while wearing a mask^11,12^. In addition, a study using a particle counter indicated that the number of particles dispersed in the direction of the eyes varied with changes in the way masks were worn^13^. A study using infrared cameras to observe temperature changes around the eyes and to visualize exhaled air reported that wearing a mask may create a jet stream toward the eyes, thereby increasing the possibility of contamination^14^. One study used the schlieren imaging technique to record airflow around the eyes while participants were wearing masks^12^. A multicenter study in Japan reported an increase in the incidence of endophthalmitis after vitrectomy following the start of the COVID-19 pandemic^15^. The same study also reported that the incidence of endophthalmitis after cataract surgery did not increase. This inconsistency can be explained by the vulnerability of the vitreous cavity to bacterial infection. An animal experiment study reported that endophthalmitis occurs upon administering few bacteria into the vitreous cavity than those administered into the anterior chamber to induce the condition^16^. It can be stated that more stringent precautions are needed during vitrectomy to avoid bacterial contamination.

These reports have discussed the possibility that airflow resulting from daily facemask use may increase the risk of endophthalmitis; however, exhaled airflow can also be directed toward the eyes during surgery. During surgery, the periocular area is completely covered by a drape. However, it is common for the adhesive portion of the drape to peel off and leave a gap between the skin and drape during long surgeries or excessive patient movement. As a result, it has been reported that airflow can be directed from the site of incomplete coverage toward the eyes^17^. Contamination of the surgical field by bacteria dispersed in exhaled airflow may increase the risk of bacterial endophthalmitis. However, no previous studies have quantitatively examined the potential of exhaled airflow to contaminate the eyes during surgery. Therefore, in this study, we aimed to examine how the risk of contamination varies depending on the state of the drapes by conducting a quantitative evaluation through the visualization of exhaled airflow using a carbon dioxide (CO_2_) imaging camera and by measuring particle counts with a particle counter under conditions simulating endophthalmic surgery.

## Results

Nine healthy adult men with a median age of 27 years (range, 24–31 years) volunteered to participate in this study.

### CO_2_ imaging

**Figure 1:**
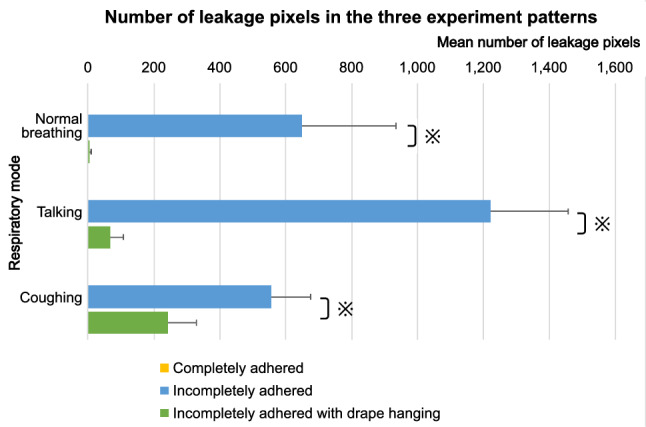
The mean number of leakage pixels corresponding to the three experiment patterns. The asterisks represent significant differences (*p* < 0.05).

Figure 1 shows the number of “leakage pixels” corresponding to the three experiment patterns. Leakage pixels were determined by averaging the number of exhaled airflow pixels in all frames. When the drape adhered entirely to the skin (experiment pattern 1), there was no leakage of exhaled airflow into the operative field, and there were zero leakage pixels in all the respiratory modes. By contrast, airflow toward the eye occurred in all respiratory modes when a quarter of the adhesive part of the drape (nasal inferior part) was peeled off (experiment pattern 2). The mean numbers of leakage pixels were 648.83, 1220.58, and 554.89 during normal breathing, talking, and coughing, respectively. Furthermore, airflow decreased in all respiratory modes when the drape was hung, creating space above the chest (experiment pattern 3). The mean numbers of leakage pixels were 45.89, 66.79, and 241.98 during normal breathing, talking, and coughing, respectively. Comparing patterns 2 and 3, significant changes in the number of leakage pixels were observed in all respiratory modes (*p* < 0.05). Supplementary Video 1 represents all the experiment patterns (image processing step “b” described in the "Methods" section).

### Particle counts

The measurement was performed for 30 s at a flow rate of 2.83 L/min, and the results were presented as the number of particles in 1.415 L. Prior to conducting the experiment, the particle count on the operating table was zero for all particle sizes, which was consistent with that in the air-conditioned operating room.

Table 1 shows the differences in particle counts by respiratory modes before and after peeling off a quarter of the adhesive part of the drape. The mean increase in total particles was measured over a 30-s period after peeling off the drape, and we found that the total particles increased by 7.33 during normal breathing (*p* = 0.63), by 71.89 during talking (*p* = 0.0039), and by 214.44 during coughing (*p* = 0.027). As for the increase by specific particle sizes, the 0.3- and 0.5-μm sized particles significantly increased during talking by 42.89 (*p* = 0.0078) and 20.44 (*p* = 0.0078), respectively. During coughing, the 0.3- and 0.5-μm-sized particles significantly increased by 149.00 (*p* = 0.031) and 57.67 (*p* = 0.027), respectively. Other combinations of particle sizes and respiratory modes showed no significant changes in particle counts after peeling the drape.

**Table 1:** Differences in particle counts by particle size and respiratory mode between experiment patterns 1 and 2.

Particle size (μm)	Respiratory mode (n = 9)	Mean difference [pattern (2)-pattern (1)]	%Change in mean particle count	Standard error of mean difference	Median of differences	Signed rank *p* value < 0.05
All	Normal	7.33	54.55%	6.05	0	No (0.63)
	**Talking**	**71.89**	**330.10%**	**22.35**	**41**	**Yes (0.0039)**
	**Coughing**	**214.44**	**652.03%**	**129.72**	**78**	**Yes (0.027)**
0.3	Normal	3.78	72.34%	2.42	1	No (0.36)
	**Talking**	**42.89**	**559.42%**	**14.35**	**27**	**Yes (0.0078)**
	**Coughing**	**149.00**	**1072.80%**	**90.12**	**58**	**Yes (0.031)**
0.5	Normal	2.33	47.73%	2.32	0	No (0.56)
	**Talking**	**20.44**	**248.65%**	**5.73**	**17**	**Yes (0.0078)**
	**Coughing**	**57.67**	**513.86%**	**34.65**	**19**	**Yes (0.027)**
1	Normal	1.22	37.93%	1.81	− 1	No (0.81)
	Talking	7.89	139.22%	3.33	6	No (0.055)
	Coughing	7.11	92.75%	5.42	1	No (0.23)
3	Normal	0.00	0.00%	0.00	0	No (1)
	Talking	0.33	150.00%	0.22	0	No (0.5)
	Coughing	0.56	500.00%	0.36	0	No (0.31)
5	Normal	0.00	Incalculable	0.00	0	No (1)
	Talking	0.33	Incalculable	0.31	0	No (1)
	Coughing	0.11	Incalculable	0.10	0	No (1)
10	Normal	0.00	Incalculable	0.00	0	No (1)
	Talking	0.00	Incalculable	0.00	0	No (1)
	Coughing	0.00	Incalculable	0.00	0	No (1)

Bold rows highlight the scenarios in which significant differences were observed.

Table 2 shows the difference in particle counts between experiment patterns 2 and 3 based on the respiratory modes. The mean decrease in total particles was measured over a 30-s period after hanging the drape and creating a space above the chest, and the results showed that total particles decreased by 19.56 during normal breathing (*p* = 0.023), by 89.33 during talking (*p* = 0.0039), and by 211.67 during coughing (*p* = 0.0078). As for the decrease by specific particle sizes, during normal breathing, the 0.3-, 0.5-, and 1.0-μm sized particles significantly decreased by 8.00 (*p* = 0.023), 7.00 (*p* = 0.031), and 4.44 (*p* = 0.016), respectively. During talking, the 0.3-, 0.5-, and 1.0-μm sized particles significantly decreased by 48.33 (*p* = 0.0039), 27.22 (*p* = 0.0039), and 12.89 (*p* = 0.0039), respectively. During coughing, the 0.3- and 0.5-μm-sized particles significantly decreased by 139.89 (*p* = 0.0078) and 59.00 (*p* = 0.0078), respectively. No other combinations of particle sizes and respiratory modes showed significant changes in particle counts.

**Table 2:** Differences in particle counts by particle size and respiratory mode between experiment patterns 2 and 3.

Particle size (μm)	Respiratory mode (n = 9)	Mean difference [pattern (3)- pattern (2)]	%Change in mean particle count	Standard error of mean difference	Median of differences	Signed rank *p* value < 0.05
All	**Normal**	**− 19.56**	**− 94.12%**	**6.60**	**− 18**	**Yes (0.023)**
	**Talking**	**− 89.33**	**− 95.37%**	**21.28**	**− 67**	**Yes (0.0039)**
	**Coughing**	**− 211.67**	**− 85.58%**	**117.82**	**− 50**	**Yes (0.0078)**
0.3	**Normal**	**− 8.00**	**− 88.89%**	**2.35**	**− 12**	**Yes (0.023)**
	**Talking**	**− 48.33**	**− 95.60%**	**13.18**	**− 35**	**Yes (0.0039)**
	**Coughing**	**− 139.89**	**− 85.88%**	**79.29**	**− 33**	**Yes (0.0078)**
0.5	**Normal**	**− 7.00**	**− 96.92%**	**2.44**	**− 5**	**Yes (0.031)**
	**Talking**	**− 27.22**	**− 94.96%**	**4.87**	**− 25**	**Yes (0.0039)**
	**Coughing**	**− 59.00**	**− 85.65%**	**32.59**	**− 18**	**Yes (0.0078)**
1	**Normal**	**− 4.44**	**− 100.00%**	**2.37**	**− 2**	**Yes (0.016)**
	**Talking**	**− 12.89**	**− 95.08%**	**3.38**	**− 9**	**Yes (0.0039)**
	Coughing	**− **12.11	**− **81.95%	6.30	**− **4	No (0.055)
3	Normal	**− **0.11	**− **100.00%	0.10	0	No (1)
	Talking	**− **0.56	**− **100.00%	0.28	0	No (0.25)
	Coughing	**− **0.56	**− **83.33%	0.36	0	No (0.31)
5	Normal	0.00	Incalculable	0.00	0	No (1)
	Talking	**− **0.33	**− **100.00%	0.31	0	No (1)
	Coughing	**− **0.11	**− **100.00%	0.10	0	No (1)
10	Normal	0.00	Incalculable	0.00	0	No (1)
	Talking	0.00	Incalculable	0.00	0	No (1)
	Coughing	0.00	Incalculable	0.00	0	No (1)

Bold rows highlight the scenarios in which significant differences were observed.

## Discussion

This is the first report to describe the exhaled airflow observed around the eye during surgery, captured with a CO_2_ imaging camera. The possibility of eye contamination resulting from airflow from the mouth first came to attention after the COVID-19 pandemic, when wearing face masks became common. Several studies were conducted to examine the possibility of increased postoperative infection due to mask-wearing^12–14^. Although these studies suggest that wearing a mask directs exhaled airflow toward the eyes, thereby causing contamination, they were not intended for surgical situations.

This study found that airflow directed toward the eye can be measured during surgery using a high-sensitivity optical gas imaging camera. Specifically, it was confirmed that when the drape tightly adhered to the skin, there was no airflow toward the eye, but once a gap was created between the drape and skin, airflow was directed toward the eye. However, even when there was a gap between the drape and the skin, the airflow was greatly reduced by hanging the drape with the help of a rihika.

The effect of drape hanging can be explained based on conductance, which has also been historically discussed in relation to breathing mechanics^18^. Patients are assumed to breathe with a constant exhalation flow rate, *Q* (L/s) under the drape. The exhaled air from the patient is discharged through many gaps (channels) that are naturally formed between the drape and the body of the patient. Conductance, *C* [L/(s Pa)], is defined as *C* = Q/ΔP, which is the ratio of the flow rate to the pressure drop, Δ*P* (Pa), between the source and exit. In a laminar flow region, which is applicable in the present case, the conductance of the individual channel is proportional to the fourth power of the effective diameter^19^. In addition, the total conductance of many parallel channels is simply the summation of the conductance of the individual channels. Therefore, if one channel with a large opening is added, the new channel acquires an overwhelmingly high conductance compared with that of the others, and accordingly, the total conductance increases drastically. Δ*P* is the pressure generated at the mouth of the patient with respect to atmospheric pressure and serves as a driving force that causes airflow through all the channels. When total conductance increases, Δ*P* decreases. Because the flow rate through each channel is proportional to individual conductance, exhaled breath is predominantly discharged through channels with high conductance. When the drape is not hung, it is in close contact with the patient's body, and small channels are naturally formed in various places. In the case of low total conductance, high Δ*P* is formed inside the drape, and the exhaled air is pushed out through individual channels with a relatively large driving force. If the drape is detached from the skin near the eye, a considerable amount of air flows out through the opening. However, if an intentional opening with a much larger size than that of the natural channels is created at the chest area by hanging the drape, Δ*P* inside the drape is remarkably reduced because of the drastic increase in total conductance. The exhaled air predominantly evacuates through the large opening at the chest in the direction of the body, and the airflow through the unsealed opening at the eye is suppressed to a negligible amount.

When the adhesion between the drape and skin was removed, the particle counter revealed that airborne particles were dispersed in the direction of the eye. Particles with sizes of 0.5 µm or 1.0 µm may contain bacteria^20^, which may cause postoperative endophthalmitis if the ocular surface becomes contaminated. In addition to contamination of the ocular surface, pathogens may adhere to the surgeon's hands and instruments. The longer the operation time, especially in vitreous surgery, the higher the likelihood of a gap forming between the drape and skin, and the airflow generated between them may cause the accumulation of contaminants.

If the ocular surface is contaminated by airflow toward the eye, a major problem that arises is that the contamination would continue for as long as the airflow is generated. Currently, iodine-based disinfectants are the standard mode of sterilizing the surgical field in ophthalmic surgery. However, in many cases, disinfection is performed only before the procedure begins. While the procedure for vitreous injections is completed quickly, endophthalmic surgery takes a much longer time, and there is a risk of ocular surface contamination during the procedure, even if the ocular surface was properly disinfected before the start of surgery. For this reason, methods, such as iodine disinfection, performed not only before the start of surgery but also during procedures with a high risk of bacterial strays, such as during lens insertion, and the application of iodine intermittently during surgery have been reported^21–23^. However, it is impractical to continuously apply antiseptics during vitrectomy procedures that use wide-angle observation lenses. In addition, if the microscope or surgeon's hands are contaminated, regardless of the number of times the ocular surface is disinfected, bacteria are likely to enter the eye. As the operative time increases, the drape often becomes incompletely adhered owing to perspiration or body movement. In such cases, it is important to keep in mind that exhaled airflow through the gap between the drape and skin can contaminate the surgical field and surgeon's hands with pathogens. Of course, it is important to apply the drapes properly so that they do not peel off and reattach them when they do. However, it is possible that the surgeon may be so focused on the surgical procedure that he or she may not notice that the adhesive has peeled off. Depending on the undulation of the patient's face and skin, the drape may inevitably and easily peel off. If the drape is hung up in advance at the beginning of surgery, even if it is removed from the skin, the amount of airflow and particles leaking into the surgical field can be reduced. Endophthalmitis caused by oral bacteria is said to have a poor prognosis, which is another reason to be particularly careful about contamination from exhaled air^10^.

This study has several limitations. One is the variability in the respiratory rate, ventilation rate, and intensity of coughing among the participants. Care was taken to minimize the effects of these variations by increasing the length of time the particle counter was used and by increasing the length of time the video was captured. The second is with regard to air conditioning. While the air conditioning needed to be on to maintain consistency between the particle measurements and the actual surgical conditions, the airflow from the air conditioning prevented clear visualization of the CO_2_ disbursement. Unfortunately, this tradeoff made it difficult to perform the two experiments under the same conditions. However, since air conditioning does not affect the airflow under the drapes, the exhaled air leaking through the gap near the eye was not likely affected by air conditioning. Therefore, the findings of this study can be applied to actual air-conditioned surgical environments. On particle counting, the low number of particles measured compared to the operating room capability may be due to the particle counters not measuring all particles present. However, all particle measurements were taken under the same conditions, and the increase in particle counts when holes were made in the drape and the decrease when the drape was rihika are believed to reflect a change in the true particle counts. There are also limitations to the image analysis methods that were used: the brightness of the airflow captured by a CO_2_ imaging camera varies depending on the concentration of CO_2_ and temperature of the exhaled air. The leakage pixels do not reflect the volume of gas leaked, and even with the same volume of airflow, the faster the velocity, the shorter the video captured, and consequently, the smaller the calculated number of leakage pixels. Therefore, it was difficult to perform comparisons between different breathing patterns. In addition, because videos were only captured in the lateral direction, the airflow leakage in the depth direction along the line of sight may have been underestimated. The establishment of three-dimensional imaging techniques of exhaled airflow based on two or more CO_2_ imaging cameras is expected. However, this method is considered sufficiently objective for comparing the same breathing patterns. No microbiological tests, such as bacterial cultures, were performed; therefore, it was not possible to determine specifically the amount of bacteria or other substances that reached the ocular surface due to airflow leakage. Above all, this study was limited to experiments conducted when drapes were used. Therefore, the findings of this study cannot be applied to how exhaled air can contaminate the ocular surface during procedures or treatments in which drapes are not used.

Maintaining cleanliness is one of the most important factors in surgery. Considering the risk of serious visual impairment once endophthalmitis occurs, it is important to take all possible precautions to maintain cleanliness. Based on the findings of this study, we recommend that the possibility of contamination of the surgical field by exhaled air currents be kept in mind during surgery and that this possibility be minimized in advance by drape hanging.

## Methods

This prospective observational cohort study was conducted in accordance with the principles of the Declaration of Helsinki. Approval was obtained from the Institutional Review Board (IRB) of the University of Fukui prior to the start of the study. The participants were required to be healthy adults between 18 and 80 years of age. Written informed consent was obtained from all participants for the study protocol and for the publication of identifying information/images in online open-access publications. This study was registered with the University Hospital Medical Information Network-Clinical Trials Registry of Japan (ID, UMIN 000047419; date of access and registration, April 6, 2022).

**Figure 2:**
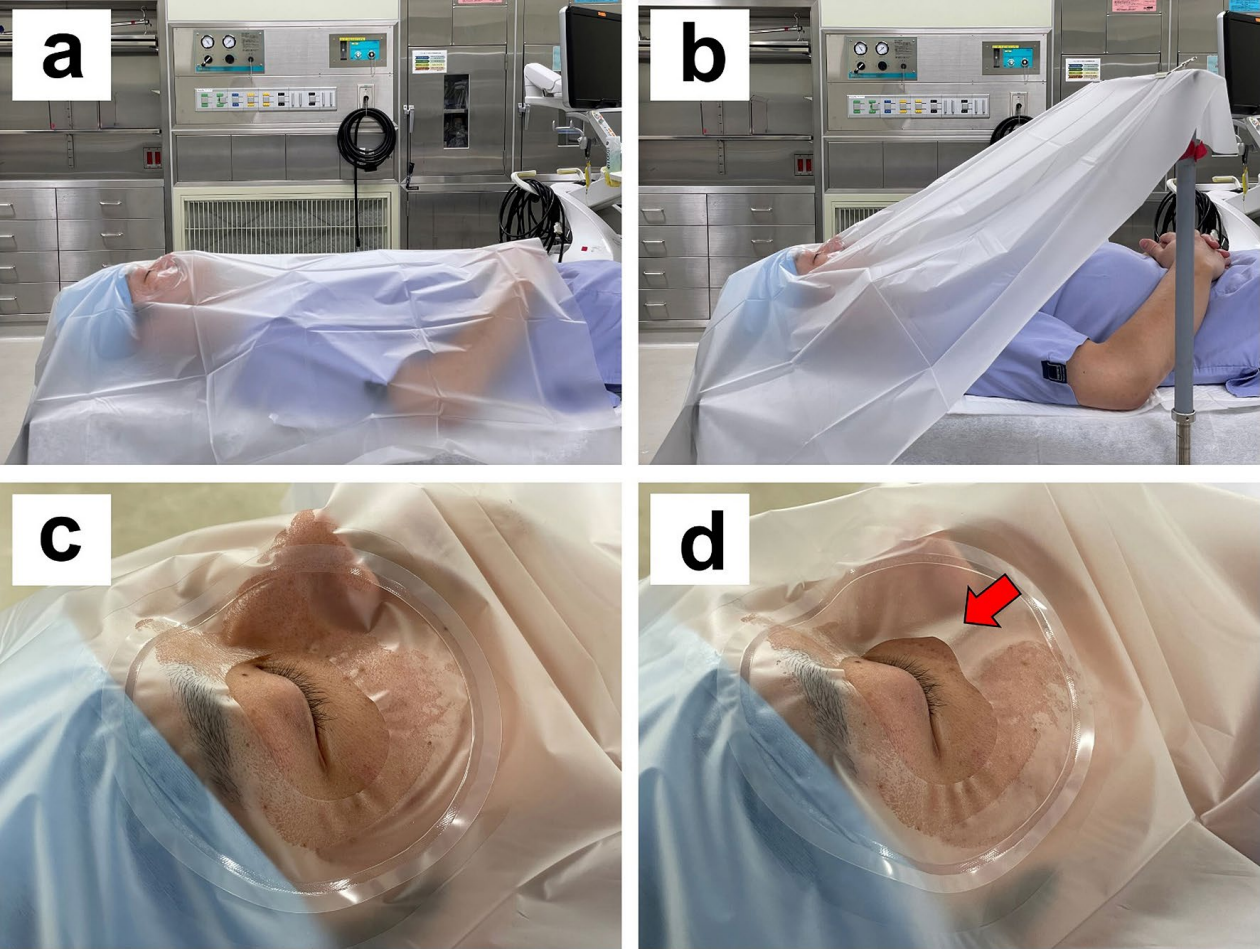
Overview of the experiment and the periocular region after draping. (**a**) The drape was not hung in experiment patterns 1 and 2. (**b**) The drape was hung with the rihika, and there was a space above the chest in experiment pattern 3. (**c**) The adhesive part of the drape completely adhered in experiment pattern 1. (**d**) A quarter of the adhesive part of the drape was peeled off (red arrow; the nasal inferior part; width, 30 mm) in experiment patterns 2 and 3.

The study was conducted in the operating room at the University of Fukui Hospital. The room had a specialized air filtration system (AIR WATER SAFETY SERVICE INC. Kobe, Japan, ISO 14644-1 Class 7, Fed. Std. 209D Class 10,000) and no open windows. The room temperature was maintained at 25 °C and 40% humidity. Each participant lay on a surgical bed, which was horizontal to the ground, simulating the surgical condition. Five investigators wearing surgical masks performed the experiment in this room without speaking. Each participant was covered with a surgical drape (eye drape: Sumitomo Bakelite Co., Ltd. Tokyo, Japan) during the experiment. The experiment was divided based on the following three patterns. In pattern 1, the adhesive part of the drape was completely attached, and in pattern 2, a quarter of the adhesive part of the drape (the nasal inferior part; width, 30 mm) was peeled off. The drape is most likely to peel off the inferior nasal area, which is less flat than the other areas. Of the two patterns, pattern 1 was performed first, followed by pattern 2. In pattern 3, the drape was hung up with a metal rod called “rihika” in addition to the condition in pattern 2. A rihika is fixed to the operating table and is usually used to create a space from the patient's mouth to the chest area to ensure the visibility of the intubation site during general anesthesia and to alleviate the constrictive feeling caused by the drape during surgery under partial anesthesia. In this experiment, a rihika was placed horizontally across the torso 200 mm above the subject's abdomen, and the end of the drape was clipped onto it. Figure 2 shows the actual experiment setting. In each pattern, the participants were asked to breathe normally, to speak normally using a standardized script, or to cough repeatedly. The order of the respiratory modes was randomized. In total, data corresponding to nine permutations of varying drape scenarios and respiratory modes were collected from each participant.

### Particle counter

We experimented based on a previous study by Schultheis et al*.*^13^*.* A Handheld Particle Counter (Model 3889, KANOMAX JAPAN Inc., Tokyo, Japan) was used to quantify the number of particles for each drape pattern and respiratory mode permutation. The particle counter was placed on the forehead of the participants, and the particles were measured 15 mm above their right eye. The particle counter quantifies the total number of particles and categorizes the particle counts by set sizes (0.3, 0.5, 1, 3, 5, and 10 μm). A flow rate of 2.83 L/min was used, and in each trial, data were collected for 30 s. As a result, the number of particles in 1.415 L was measured. After each drape trial, the particles were allowed to diffuse during a 30-s interval. After completing all the trials in one participant, a 5-min interval was allowed before starting the trials in the next participant. The particle counts were zeroed before each new drape scenario to avoid counting the sum of particles in the previous trials. To measure the number of particles dispersed under actual surgical conditions, measurements with the particle counter were performed with the air conditioning in the operating room turned on.

### CO_2_ imaging camera

An A6796 CO_2_ imaging camera (Teledyne FLIR LLC, Wilsonville, OR, USA) was used to visualize the exhaled breath. This is a high-sensitivity custom-made camera developed for optical gas imaging in the case of leakages or for imaging the flow of CO_2_. Our camera is superior to commercial products from the same manufacturers mainly in terms of resolution, lens compatibility, and flexibility in frame rate and exposure settings. This allows us to capture images under the conditions required for experiments. This camera combines a bandpass filter tailored to the CO_2_ molecules with an absorption/emission wavelength centered at 4.26 μm with an indium antimonide image sensor with 640 × 512 pixels. By cooling both the image sensor and bandpass filter to a cryogenic temperature to suppress thermal emission from the filter, the visualization of CO_2_ gas with high contrast is achieved.

The camera was fixed horizontally onto a tripod, and the area around the head of the participant was photographed from the right side through an infrared lens (focal length: 25 mm, F-number: 2.5). A black body board (emittance: 0.97, temperature: 23 °C) was placed to the left of the participant to obtain a uniform background and to avoid capturing anything apart from the participant and airflow generated by the participant in the video. To avoid noise in the video images caused by airflow from the air conditioning, the video was shot with the air conditioning turned off. The air conditioning was turned off for imaging approximately 10 min per examinee, and CO_2_ concentration was maintained at a constant level (440–482 ppm during the experiment). Video images were recorded with an exposure time of 40 ms at 25 frames/s.

In optical gas imaging techniques, gas is observed as bright or dark smoke^24^. The intensity of visualizing the gas is determined by the relationship between the temperature of the gas and the background; cold CO_2_ appears dark, but hot CO_2_ appears bright. In this study, exhaled breath was visualized with a higher intensity than the background because hot CO_2_ at a temperature close to that of the body was observed in front of the black body board at room temperature. Because the concentration of CO_2_ in exhaled air is higher (typically 4%) than that in ambient air (typically 400 ppm), the camera selectively visualizes exhaled air.

### Image processing

**Figure 3:**
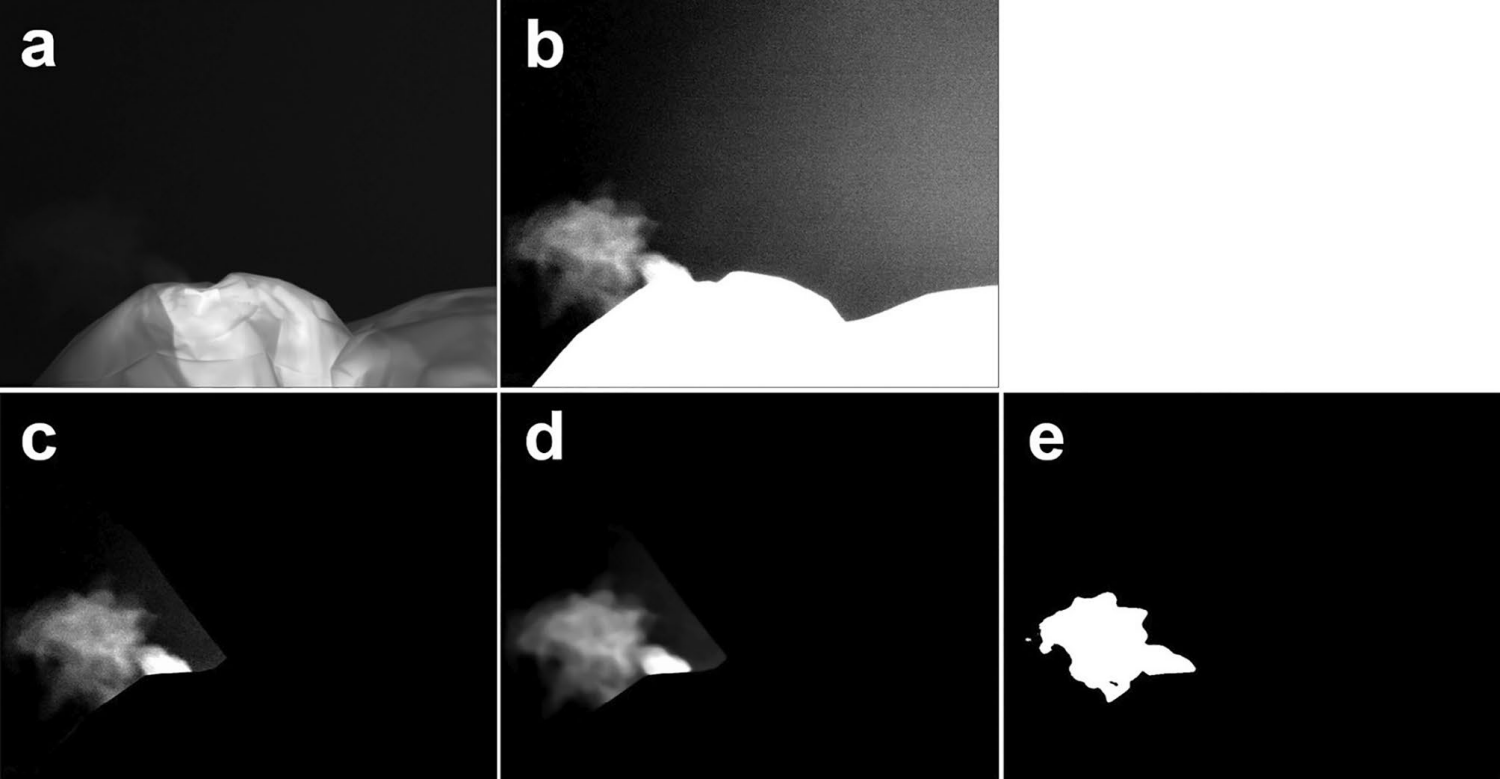
Five steps involved in image processing. (**a**) Unprocessed image. (**b**) Image with dynamic range and contrast adjusted for airflow. (**c**) Image with masked non-airflow areas. (**d**) Image with noise reduction and smoothing. (**e**) Binarized image.

The video recorded by the infrared camera was adjusted using FLIR ResearchIR MAX software (Teledyne FLIR LLC, which aids in obtaining images with a dynamic range and contrast adjusted for airflow; this enables the clearest possible visualization of the airflow shape. Subsequently, the non-airflow areas in the images were masked using Adobe Premiere Pro 2022 (Adobe, San Jose, CA, USA), which was followed by denoising and smoothing in MATLAB 2021b (MathWorks, Natick, MA, USA). Finally, the images were binarized, and the number of pixels in the airflow areas was evaluated in each frame. The total number of target pixels in the entire video was divided by the number of frames in the video, and the resulting number of leakage pixels was used as an indicator of airflow leakage. Figure 3 shows the steps involved in image processing. The National Institute for Materials Science (NIMS) and Photron Limited assisted with the filming and provided advice on video analysis.

### Statistical analysis

The Wilcoxon signed-rank test was used to test for paired differences in the number of leakage pixels between patterns 2 and 3 and in the particle counts in each respiratory mode. Paired differences in particle counts between patterns 1 and 2 and between patterns 2 and 3 were calculated for each respiratory mode and each particle size. All statistical analyses were performed using MATLAB 2021b (MathWorks, Natick, MA, USA). Statistical significance was defined as a two-sided *p* value < 0.05.

## Supplementary Information

Supplementary Video 1.

## Data Availability

The datasets generated and/or analyzed during the current study are available from the corresponding author upon reasonable request.
